# Perioperative Bilateral Medial Medullary Infarction With “Snake Eyes Appearance”: A Case Report

**DOI:** 10.3389/fmed.2021.559381

**Published:** 2021-09-09

**Authors:** Yicheng Xu, Ke Li, Xinlu Yao, Qiyan Yang, Peifu Wang

**Affiliations:** ^1^Department of Neurology, Aerospace Center Hospital, Beijing, China; ^2^Department of Neurology, Peking Union Medical College Hospital, Chinese Academy of Medical Sciences and Peking Union Medical College, Beijing, China; ^3^Department of General Practice, Zhongshan Hospital, Fudan University, Shanghai, China

**Keywords:** perioperative care, stroke, coronary artery bypass, atherosclerosis, Guillain-Barre syndrome, magnetic resonance imaging, case report

## Abstract

Perioperative bilateral medial medullary infarction (BMMI) cases mimicking acute motor axonal neuropathy (AMAN) under general anesthesia have not been reported. We describe a patient who suffered flaccid quadriplegia and could not wean from mechanical ventilation after emergence from general anesthesia in cardiac surgery. A diagnosis of AMAN was considered, but intravenous immunoglobulin showed little efficacy. Magnetic resonance imaging of the patient later revealed BMMI with “snake eyes appearance,” and he was found to have severe vertebral artery stenosis. Considering the association between severe coronary heart disease and cerebrovascular stenosis, we highlight the significance of preoperative evaluation and comprehensive management of the cerebrovascular system for certain patients.

## Introduction

Bilateral medial medullary infarction (BMMI) is a rare but devastating subtype of cerebrovascular accident that may lead to acute-onset quadriplegia and respiratory failure (1). Accordingly, it is clinically significant to differentiate between BMMI and acute motor axonal neuropathy (AMAN), a subtype of Guillain-Barré syndrome (GBS), due to their similar clinical manifestations. Although cerebrospinal fluid tests, electromyography, and magnetic resonance imaging (MRI) may be helpful for differentiating BMMI and AMAN, results of those tests can be ambiguous at times. Additionally, MRI may be unfeasible for perioperative patients under mechanical ventilation.

Ischemic stroke is a devastating perioperative complication, especially for patients undergoing cardiac surgery. According to a recent study, ~1.6% of the patients undergoing coronary artery bypass grafting (CABG) suffered postoperative stroke (2). Patients with severe coronary artery stenosis often have concomitant carotid or vertebral artery stenosis (3, 4), which may be significant etiologic factors for post-CABG stroke (5). In an attempt to prevent post-CABG stroke, current published guidelines attach much importance to the preoperative management of stenotic carotid arteries (6), but few of them focus on stenotic vertebral arteries and the potentially related posterior circulation stroke which may be as well-clinically important.

Herein, we present a unique post-CABG BMMI case manifesting as AMAN with quadriplegia, respiratory failure, and severely stenotic vertebral arteries. MRI of the patient showed an unusual “snake eyes appearance (SEA)” lesion distinct from the typical heart-shaped lesion in BMMI patients (1). Additionally, relevant diagnostic challenges, possible pathophysiological mechanisms, and implications for perioperative management have also been discussed.

## Case Report

A 67-year-old Asian man was admitted into our institution, a tertiary comprehensive hospital, because of quadriplegia after off-pump CABG. Seventeen days before admission, he underwent CABG in a hospital specializing in heart diseases due to severe stenosis of the left main coronary artery and occlusion of the right coronary artery, but suffered acute respiratory failure and quadriplegia after emergence from general anesthesia. His blood pressure was 100/50 mmHg, heart rate 116 beats per minute, and respiratory rate 24 breaths per minute. Physical examinations of the heart and lung was unremarkable. Neurological examination revealed flaccid quadriplegia (muscle strength of all limbs at 0/5) with intact sensory and oculomotor function as well as negative Babinski signs. Computed tomography (CT) scan within 1 day after onset excluded the possibility of intracranial hemorrhage, but head MRI was considered unfeasible because the patient was critically ill under mechanical ventilation and life support equipment along with ventilators compatible with MRI were not available in the MRI room. Tracheotomy was performed 8 days after onset of quadriplegia to facilitate ventilation. Despite proactive management, the patient showed little improvement, after which was transferred to our hospital for further treatment.

The patient had a 10-year history of hypertension and was found to have coronary heart disease 2 months ago, but denied histories of diabetes mellitus, myocardial infarction, atrial fibrillation, stroke, smoking, or alcohol abuse. On admission, blood pressure of the patient was 146/72 mmHg, and other vital signs were within normal range. He was alert but not able to speak due to mechanical ventilation. Neurological examination was otherwise normal except for quadriplegia, disturbance of bilateral soft palate elevation, and weakness of head turning, without nystagmus, sensory loss, positive Babinski signs, or palsy of cranial nerves III, IV, VI, VII, and XII. The laboratory tests showed white blood cell of 9.49/μL, hemoglobin of 8.4 g/dL, and creatinine of 1.03 mg/dL, with normal serum sodium, potassium, and low-density lipoprotein cholesterol levels. His electrocardiogram and chest X-ray were unremarkable.

**Figure 1:**
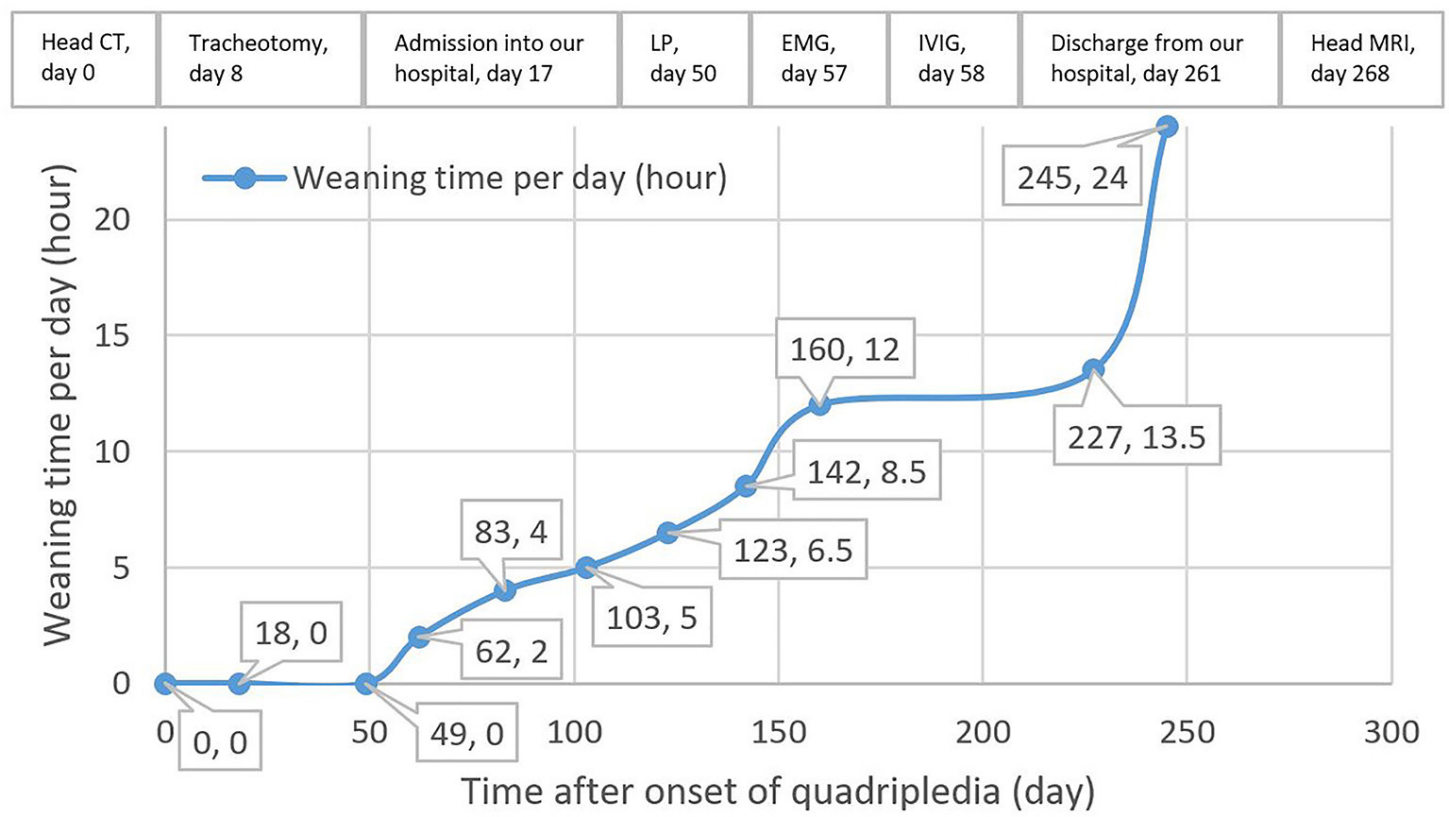
Weaning time and timeline of the management of the patient after onset of quadriplegia. CT, computed tomography; LP, lumbar puncture; EMG, electromyography; IVIG, intravenous immunoglobulin; MRI, magnetic resonance imaging.

**Figure 2:**
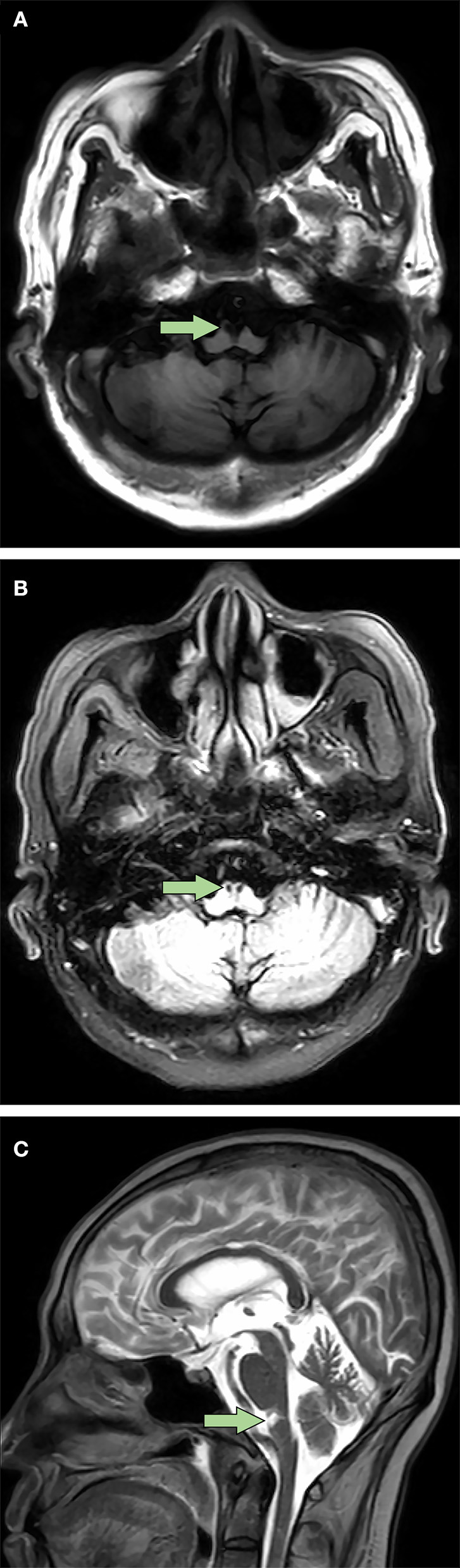
Bilateral medial medullary infarction with “snake eyes appearance” in magnetic resonance imaging 9 months after onset. **(A)** Axial T1-weighted images showed a typical heart-shaped lesion with low signal intensity at ventral rostral medulla (arrow). **(B)** Different from the axial T1-weighted images, axial T2-weighted fluid-attenuated inversion recovery images showed bilateral lesions with low signal intensity which presented as a special “snake eyes appearance” at the same location (arrow). **(C)** Sagittal T2-weighted images revealed similar lesions with high signal intensity at ventral rostral medulla (arrow).

Postoperative acute-onset flaccid quadriplegia and respiratory failure without positive Babinski signs and sensory dysfunction suggested the diagnosis of AMAN. A week after admission, findings from his cerebrospinal fluid (CSF) showed a slightly elevated protein level of 0.4749 g/L (reference range 0.1500–0.4500 g/L) with normal white blood cell count. Furthermore, electromyography mainly suggested axonal damage of motor nerves (see Supplementary Table 1), which further confirmed the diagnosis. Therefore, intravenous immunoglobulin was promptly administered at 0.4 g/kg per day for 4 days, with little observed efficacy. Throughout his hospitalization, the patient remained quadriplegic. Left-side muscle strength of the patient fluctuated between 1/5 and 2/5 and the right side between 0/5 and 1/5. With intermittent weaning training (Figure 1), respiratory function of the patient gradually improved. As a result, he managed to wean from mechanical ventilation 8 months after admission, which allowed him to be transferred to a rehabilitation institution. However, his axial T2-weighted fluid-attenuated inversion recovery images 9 months after onset of quadriplegia showed lesions with low signal intensity presenting as SEA at rostral medulla level; moreover, axial T1-weighted images revealed similar lesions with low intensity at the same location (Figure 2). These findings indicated that the patient actually suffered BMMI.

After reviewing the patient's medical records thoroughly, we found several unnoticed histories of his which clearly supported the diagnosis of BMMI. Preoperative ultrasonography of the patient suggested 41% stenosis of the right common carotid artery and mild to moderate stenosis of the left one (exact percentage of stenosis unknown), with complete occlusion of the right vertebral artery and 71% stenosis of the left one. Postoperative head CT revealed multiple old lacunar infarct, a consistent predictor of post-CABG stroke (7). Intraoperative hypotension detected by invasive blood pressure monitoring via a radial artery catheter and a postoperative blood transfusion history of the patient were also noticed. Nevertheless, echocardiography and electrocardiography results of the patient showed no signs of perioperative cardiac arrhythmia, patent foramen ovale, valvular diseases, cardiomyopathy, or ventricular aneurysm.

Finally, a diagnosis of BMMI instead of AMAN was made. His moderately elevated CSF protein level could be considered normal, as a systematic review showed that the upper reference limit values of CSF protein incrementally exceeded 0.60 g/L after age 50 (8). Abnormal electromyography findings of the patient might be attributed to diabetic peripheral neuropathy, because his blood glucose levels during hospitalization met the diagnostic criteria for diabetes mellitus.

## Discussion

### Diagnostic Challenges of the Case

In recent years, only Searcy et al. emphasized the necessity to rule out motor-variant GBS in BMMI patients (9). To our knowledge, we are the first to describe a case of an AMAN-like BMMI patient in a perioperative setting. In our case, it is difficult to exclude the diagnosis of AMAN for the following reasons. Firstly, known as the most common GBS subtype in Asia, AMAN features pure motor deficits and a short interval from onset to nadir, which are in accordance with the characteristics of our patient. Secondly, operations may induce the onset of AMAN (10). Finally, the diagnosis and management of stroke become more challenging after surgery and general anesthesia, largely because of the difficulties to access MRI as mentioned above and the increased risk of surgical-site hemorrhage after thrombolysis for perioperative patients (11). As a result, clinicians may tend to diagnose a more treatable condition instead, such as AMAN.

### Implications for the Management in Preoperative Patients

Atherosclerosis is a systemic condition; thus, carotid or vertebral artery stenosis often coexist with severe coronary heart disease (3, 4), as is the case for our patient. For patients with carotid stenosis scheduled to undergo CABG, a clear management strategy has been established. According to recent guidelines regarding CABG, preoperative carotid screening by duplex ultrasound may be recommended for patients with multivessel coronary artery disease, carotid bruit, or a recent history of stroke (12), and prophylactic carotid revascularization may be considered for patients with severe bilateral carotid lesions or previous stroke histories (6). However, in remarkable contrast to the circumstance for carotid stenosis, few recommendations are available for the evaluation and treatment of severe vertebral artery stenosis in perioperative patients. One may criticize that vertebral artery stenosis may just be a surrogate marker for diffuse atherosclerotic disease rather than a direct etiologic factor in patients with perioperative stroke (13). Nevertheless, in a study involving 407 patients with posterior circulation stroke or transient ischemic attack, vertebrobasilar or posterior cerebral artery occlusive lesions caused hemodynamic cerebral infarction in 32% of the patients (14). Additionally, vertebral artery stenosis in our patient was so disproportionately severe that it might serve as an etiologic factor of post-CABG posterior circulation stroke, instead of just a marker for the general atherosclerotic burden, especially when intraoperative hypotension and impaired cerebral autoregulation caused by previous lacunar stroke (15) were present. This assumption was probably correct because stroke of the posterior circulation occurred in our patient after CABG. Stenosis of vertebral arteries stimulates clot formation and microembolization, which, combined with hypoperfusion, may lead to stroke because of decreased embolus washout (16). Although thromboembolic stroke after cardiac surgery in the posterior circulation might increase the chance of cardiac origins (17), echocardiogram and electrocardiogram of the patient were basically normal, which ruled out the possibility of cardiogenic stroke.

Our case suggests that preoperative evaluation of the cerebrovascular system (especially the posterior circulation which was often overlooked) using ultrasonography might be necessary for senior patients with severe coronary stenosis about to undergo elective CABG. For those with severe vertebral artery stenosis and histories of ischemic stroke, comprehensive risk reduction strategies including staged or synchronous revascularization of vertebral arteries and CABG might be considered on a case by case basis, and intraoperative hemodynamic monitoring, for instance, via transcranial doppler, near-infrared spectroscopy (18), or other systems, should be emphasized, so as to prevent the devastating postoperative ischemic stroke of the posterior circulation. Further randomized trials and other studies are required to figure out the necessity and strategies of preoperative management and intraoperative monitoring for patients scheduled to undergo elective CABG.

### The “Snake Eyes Appearance” in Magnetic Resonance Imaging

Distinct from the typical heart-shaped appearance in MRI (1), the special SEA-like lesion in BMMI patients has yet been previously reported. Traditionally described as two symmetric hyperintense spots in the spinal cord in T2-weighted MRI images, SEA is a special radiological finding reported in a variety of heterogeneous diseases, including spontaneous spinal ischemia (19), amyotrophic lateral sclerosis (20), and cervical spondylotic myelopathy (21). Ischemia is one of the proposed underlying mechanisms of SEA (21). The SEA-like lesion in our patient was presumably caused by infarction in the distal branches of the vertebral arteries or basilar artery that supplied bilateral pyramidal tracts. Our case showed for the first time an SEA-like lesion at rostral medulla level in patients with BMMI, while SEA in previously reported cases primarily located in the gray matter from C4 to T6 segments of the spinal cord (19–21).

## Concluding Remarks

In summary, the case of our patient with BMMI misdiagnosed as AMAN after CABG with a special SEA-like lesion in MRI might provide several novel perspectives on the prevention and diagnosis of perioperative ischemic stroke of the posterior circulation in senior patients.

## Data Availability Statement

The raw data supporting the conclusions of this article will be made available by the authors, without undue reservation.

## Ethics Statement

Written informed consent was obtained from the individual(s) for the publication of any potentially identifiable images or data included in this article.

## Author Contributions

YX and KL drafted the manuscript. YX, KL, and QY collected patient information, searched literature, and prepared the figure. YX, XY, and PW evaluated and managed the patient. PW interpreted the data and edited the manuscript finally. All authors contributed to the article and approved the submitted version.

## Conflict of Interest

The authors declare that the research was conducted in the absence of any commercial or financial relationships that could be construed as a potential conflict of interest.

## Publisher's Note

All claims expressed in this article are solely those of the authors and do not necessarily represent those of their affiliated organizations, or those of the publisher, the editors and the reviewers. Any product that may be evaluated in this article, or claim that may be made by its manufacturer, is not guaranteed or endorsed by the publisher.
