# Supplementary material for: Perioperative Bilateral Medial Medullary Infarction With “Snake Eyes Appearance”: A Case Report
Source: Front Med (Lausanne). 2021 Sep 9;8:559381. doi: 10.3389/fmed.2021.559381 (PMC8458652; doi:10.3389/fmed.2021.559381)
Supplement: Supplementary file 1 [file Table_1.DOCX]

Supplementary Material

# Supplementary Table 1. Electromyography results of the patient after admission to our hospital.

| **Motor Nerve Conduction** | | | | | | | | | | |
| --- | --- | --- | --- | --- | --- | --- | --- | --- | --- | --- |
| **Nerve** | **Stimulation Site** | | **Lantency (ms)** | **Amplitude (mV)** | | **Segment** | **Lantency Difference (ms)** | | **Distance (mm)** | **Conduction Velocity (m/s)** |
| **Median (R)** | Wrist | | 3.6 | 4.6↓ | | Wrist to APB | 3.6 | | / | / |
|  | Elbow | | 8.2 | 4.4↓ | | Elbow to wrist | 4.6 | | 240 | 52 |
| **Ulnar (R)** | Wrist | | 3.1 | 2.9↓ | | Wrist to ADM | 3.1 | | / | / |
|  | Below elbow | | 8.4 | 1.5↓ | | Below elbow to wrist | 5.3 | | 280 | 53 |
| **Peroneal (R)** | Ankle | | 4.7 | 0.5↓ | | Ankle to EDB | 4.7 | | / | / |
|  | Fibular head | | 10.3 | 0.6↓ | | Fibular head to ankle | 5.6 | | 310 | 55 |
| **Tibial (R)** | Ankle | | 5.7 | 0.1↓ | | Ankle to AH | 5.7 | | / | / |
|  | PF | | NA | NA | | PF to ankle | NA | | NA | NA |
| **Sensory Nerve Conduction** | | | | | | | | | | |
| **Nerve** | **Stimulation Site** | | **Lantency (ms)** | **Amplitude (μV)** | | **Segment** | **Lantency Difference (ms)** | | **Distance (mm)** | **Conduction Velocity (m/s)** |
| **Median (R)** | Index finger | | NA | NA | | Index finger to wrist | NA | | NA | NA |
| **Ulnar (R)** | Fifth finger | | 2.5 | 1↓ | | Fifth finger to wrist | 2.5 | | 110 | 43↓ |
| **Sural (R)** | Lower leg | | 2.6 | 4 | | Lower leg to ankle | 2.6 | | 120 | 47 |
| **F Wave Studies** | | | | | | | | | | |
| **Nerve** | | **M Wave Latency (ms)** | | | **F Wave Average Latency (ms)** | | | **F Wave Occurrence Rate (%)** | | |
| **Median (R)** | | 3.7 | | | 29.6 | | | 25.0↓ | | |
| **Ulnar (R)** | | 3.3 | | | NA | | | 0.0↓ | | |

ADM = adductor digiti minimi; AH = abductor halluces; APB = abductor pollicis brevis; EDB = extensor digitalis brevis; NA = not available because the potential was not elicited; PF = popliteal fossa; R = right.

Values below the normal range are marked with a downward arrow, and values above the normal range are marked with an upward arrow.
